# Recurrent Stress Cardiomyopathy During COPD Exacerbation: Are Beta-adrenergic Agonists Only to Blame?

**DOI:** 10.7759/cureus.1166

**Published:** 2017-04-14

**Authors:** Ioanna Katsa, Panagiota Christia, Daniele Massera, Robert Faillace

**Affiliations:** 1 Medicine, North Bronx Health Network Jacobi Medical Center; 2 Cardiology, Montefiore Medical Center; 3 Cardiology, North Bronx Health Network Jacobi Medical Center

**Keywords:** stress cardiomyopathy, takotsubo cardiomyopathy, copd exacerbation, bronchogenic takotsubo, heart failure

## Abstract

Takotsubo cardiomyopathy (TCM) is a variant of stress-induced cardiomyopathy, characterized by transient left ventricular dysfunction that may be associated with emotional or physical triggers. We present the case of a 51-year-old Caucasian female with severe chronic obstructive pulmonary disease (COPD) who presented with syncope and was found to have her second lifetime episode of stress-induced cardiomyopathy. Eight months prior, she had been admitted with a COPD exacerbation and was found to have left ventricular (LV) dysfunction with ejection fraction (EF) of 22% attributed to TCM with subsequent normalization of her left ventricular function. Recurrence of stress-induced cardiomyopathy associated with COPD is a rare phenomenon and its presentation raises the possibility of a common underlying mechanism.

## Introduction

Takotsubo cardiomyopathy (TCM) associated with chronic obstructive pulmonary disease (COPD) is considered a subset of TCM, suggesting a dangerous liaison of the heart-lung axis [1-3]. Manfredini, et al. reported a high prevalence of lung pathologies, including asthma, COPD, and pulmonary embolism in a retrospective analysis of patients suffering from TCM [2]. A 44% prevalence of asthma or COPD has been described in patients with TCM [4]. Furthermore, several case reports of TCM in the setting of inhaled beta-agonist use have been published [5-10]. Though poorly understood, the overuse of beta-adrenergic stimulators inducing sympathetic activation has been suggested as the main pathophysiologic mechanism. Recurrence of TCM is rare and has been found to have an annual occurrence rate of 1.5 - 2.9% [11]. This common association with COPD raises the concern of whether COPD is an actual risk factor for recurrence of TCM.

## Case presentation

**Figure 1:**
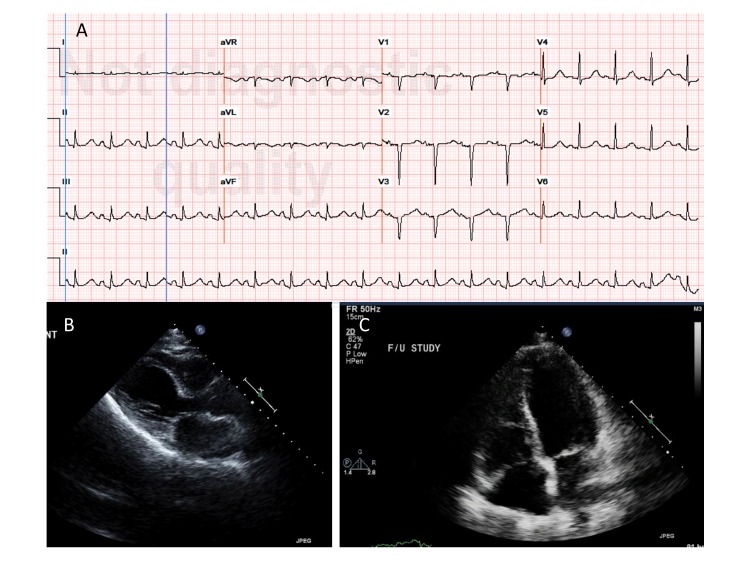
Basic cardiac evaluation. (A) Patient’s admission electrocardiogram revealed sinus tachycardia with PR depression on leads II, III, aVF, Q waves on leads V1-V3 and repolarization abnormalities. (B) A parasternal long axis view of patient's initial echocardiogram during the first episode of takotsubo cardiomyopathy. There is prominent apical ballooning of the left ventricle (LV). (C) On repeat echocardiogram, an apical four chamber view revealed resolution of the apical ballooning of the left ventricle (LV).

A 51-year-old woman, active smoker, presented to the emergency room with respiratory failure after she was found unresponsive in the bathroom. Her past medical history included severe COPD, prior alcohol and opioid dependence on methadone, hepatitis C, human immunodeficiency virus (HIV) on highly active antiretroviral therapy (HAART) with a CD4 (cluster of differentiation 4) count >300 cells/mm^3^, and an undetectable viral load. Eight months prior to admission, she was admitted with a COPD exacerbation in the setting of an influenza infection. At that time, she was found to have transient left ventricular (LV) dysfunction with an LV ejection fraction (LVEF) of 22% and apical ballooning on transthoracic echocardiogram (Figure 1B) that returned to normal (61%) five days later (Figure 1C). During her current presentation, the patient was admitted with altered mental status, responding only to painful stimuli, and severe respiratory distress with minimal air entry. She was intubated and required a brief course (24 hours) of norepinephrine intravenous infusion.

Her electrocardiogram (EKG) revealed sinus tachycardia, PR segment depression as well as repolarization abnormalities on inferior leads, and Q waves in leads V1 - V3 (Figure 1A) without subsequent dynamic changes. Cardiac biomarkers were found to be elevated (peak troponin 3.8 ng/mL, creatinine phosphokinase 1827 U/L). Her white blood cell count was elevated at 13.1 cells/mcL with a band count of 1%. Urine toxicology screen was negative for cocaine. An echocardiogram on admission revealed severely reduced LV systolic function with an LVEF of 22% and severe diffuse LV hypokinesis. After intubation, the patient received norepinephrine only on the first day of admission, and she was treated as a COPD exacerbation with intravenous steroids, albuterol, and Atrovent inhalers. She was given aspirin, clopidogrel, a high-intensity statin. She was also started on anticoagulation with a heparin drip, which was discontinued after cardiac catheterization. A beta-blocker was not administered, given her decompensated pulmonary status. On repeat echocardiogram three days later, the LV dysfunction had resolved. Coronary angiography revealed non-obstructive coronary artery disease. The patient was successfully extubated on the second day of admission.

## Discussion

Takotsubo cardiomyopathy (TCM) or stress-induced cardiomyopathy is named after a Japanese octopus trap due to the characteristic apical ballooning morphology and was first described in Japan in the early 1990s by Sato, et al. [12]. Nevertheless, stress-induced cardiomyopathy was noted a decade earlier on an autopsy study [13]. TCM is characterized by a reversible left ventricular dysfunction that is unrelated to obstructive coronary disease, valvulopathy, pheochromocytoma, or myocarditis and most commonly presents as apical ballooning. Four different types of wall motion abnormalities have been described (apical, midventricular, basal, and focal) [14-16]. TCM occurs predominately in menopausal women (mean age: 66.8 years) [14] and is associated with emotional or physical stressors. In one-third of the cases, no trigger can be identified [14, 16-17]. TCM is diagnosed in 1 - 2% of acute coronary syndrome presentations [18].

Over the last few years, a few cases of TCM have been associated with COPD or asthma exacerbations. “Bronchogenic TCM” is considered a specific form of TCM. Even though the association of lung disease with stress cardiomyopathy is well established, it remains difficult to ascertain the underlying mechanism.

With catecholamines playing a key role to the pathogenesis of the disease, beta-adrenergic stimulation appears to be a potential trigger [19-20]. Paradoxical bronchospasm induced by ipratropium has also been proposed as a possible underlying mechanism in TCM associated with COPD exacerbations [21]. It has been speculated that during COPD exacerbations there is a disproportionate predominance of sympathetic over parasympathetic activity, which could trigger TCM.

Moreover, since the early 90s, Mori, et al. suggested a very high density of beta-adrenergic receptors in the apical myocardium on a dog animal model [22]. More interestingly, Lyon, et al. described a direct catecholamine-mediated stunning effect of epinephrine on myocardial tissue [23]. Epinephrine was proposed to have myocardial cell stunning properties via inducing beta two receptors which were found to be in plethora in the apical myocardium, the main area involved in stress cardiomyopathy; it is was postulated that various forms of Takotsubo cardiomyopathy are simply the result of a different regional distribution of beta-adrenergic receptors.

Beta-agonists have been associated with increased cardiovascular morbidity and mortality in COPD patients [24]. It was believed that beta-blockers could potentially improve outcomes in TCM; however, a recently published study by the International TCM Registry found no benefit in one-year mortality in patients receiving beta-blockers [14]. During a COPD exacerbation, physical stressors such as hypoxemia and acidosis may contribute to the development of TCM. Hence, isolating the effect of beta-blockers on this sequence of events might not be the only focus [2]. 

‘Bronchogenic Takotsubo’ appears to have many subgroups and variable presentations [25]. In some cases, chest pain and a clinical picture resembling an acute coronary syndrome have been described [26-27], while in other cases, a subtle worsening of respiratory status presenting more as a pulmonary disease was reported [28]. The presence of emotional or physical triggers was also not a consistent finding among published cases.

Recurrent TCM is known and well described [29-30] and has been estimated to occur rarely with an annual rate of 1.5 - 2.9% [11] or 1.8% per patient-year [14]. It is often linked to variable conditions with most common culprits involving excessive emotional or physical stress and seizures [31], while its prevalence in patients with neurologic and psychiatric diseases is as high as 50%. On review of the literature, we found two cases of recurrent TCM during COPD exacerbations and status asthmaticus, respectively; these cases were again associated with excess adrenergic agonistic-agonist inhalation [26-27, 32].

To our knowledge, our case is the second in the literature describing recurrent TCM during COPD exacerbation [27] that was not associated with increased use of inhaled beta-agonists. It is also the first reported case of recurrent stress cardiomyopathy syndrome associated with COPD exacerbation presenting as syncope. This could suggest a multifactorial underlying mechanism, which involves the heart-lung axis rather than just an exogenous treatment.

This new subset of ‘bronchogenic Takotsubo’, despite the aforementioned speculations, remains of enigmatic pathophysiology. Cases of COPD or asthma-induced TCM are not quite common, and establishing causality by case report and case series appears unfeasible. Investigating the pathogenetic mechanisms of this rare clinical condition appears challenging. Treatment options could significantly differ considering that COPD exacerbation is treated with inhaled beta-adrenergic agonists, which are considered as a potential trigger of TCM. It also remains to be investigated whether COPD actually confers a risk of recurrence of TCM and if this recurrence could be somehow prevented or predicted.

As mentioned above, TCM and apical ballooning were considered to be related to the predominance of beta-receptors in the apical myocardium [23]. In our case report, the same patient suffered an episode of TCM with the typical apical ballooning pattern but had a recurrence of stress-induced cardiomyopathy that was characterized by diffuse hypokinesis. It needs to be underscored that this is the first reported recurrence of TCM presenting with a different pattern (apical vs diffuse) and was noted during a COPD exacerbation. This change in pattern is unique since it cannot be explained by the predominance of beta-adrenergic receptors in the apical myocardium and could potentially question the role of beta-adrenergic agonists as the main culprit on ‘bronchogenic’ stress-induced cardiomyopathy.

## Conclusions

In conclusion, this very rare recurrence of stress cardiomyopathy with two different patterns during COPD exacerbation yields many questions. Even though beta-adrenergic agonist overuse remains a reasonable hypothesis and it has been proposed as the likely pathophysiologic mechanism, there seem to be more paths to investigate. If there is any predisposition to recurrence that relates to lung disease or if this contingency is merely the result of an intrinsic inflammatory reaction during COPD remains to be explored. The pattern of distribution of beta-adrenergic receptors in the myocardium as the main mechanism of TCM morphology does not appear to explain the different patterns of stress-induced cardiomyopathy in our case.
